# Editorial: Genetic obesity

**DOI:** 10.3389/fendo.2023.1349582

**Published:** 2024-01-04

**Authors:** Maurizio Delvecchio, Graziano Grugni, Jennifer Wootton Hill

**Affiliations:** ^1^ Department of Biotechnological and Applied Clinical Science, University of L’Aquila, L’Aquila, Italy; ^2^ Division of Auxology, Istituto Auxologico Italiano, Istituto di Ricovero e Cura a Carattere Scientifico (IRCCS), Verbania, Italy; ^3^ Center for Diabetes and Endocrine Research, University of Toledo College of Medicine, Toledo, OH, United States

**Keywords:** obesity, syndromic obesity, prader-willi syndrome, body fat, colon cancer, gene-environment interaction, mendelian randomization

Obesity represents a worldwide epidemic with a significant burden for health (1). It is a complex disease, acting as a modifiable risk factor for other non-communicable diseases. Even if most cases are labelled as “idiopathic” or due lifestyle factors, research over the last two decades has shown genetic pathways also play a key role.

Monogenic obesity is a severe early-onset and rare form of obesity featuring endocrine disorders. The most frequent cause are mutations in the genes of the leptin/melanocortin axis involved in food intake regulation. Syndromic obesity is defined by severe obesity before the age of 6, accompanied by additional features like intellectual disability and organ-specific diseases. In this context, research focusing on the interaction between genetic background and environment is a growing field of research.

This Research Topic includes high-quality papers focusing on the effects of gene-environment interactions on obesity traits in low and middle-income countries, the clinical aspects of syndromic obesity, the genetic connections between obesity and colon cancer in females, the use of Mendelian randomization (MR) analysis to determine the relationship between metabolic syndrome (MetS) and cholelithiasis, and the gender-specific genetics of body fat percentage. Six papers, five original research papers, and one systematic review, were published.

Obesity has risen more in low/middle-income countries than in high-income countries (2), so Pledger and Ahmadizar examined how environmental factors such as dietary habits, physical activity, and obesity contribute to this trend. In their systematic review, they selected 13 studies using a candidate gene approach and 5 studies using a genetic risk score studies. Only one paper was a genome-wide association study. Most of the papers investigated the diet-gene and physical activity-gene interactions, but others explored the role of other factors such as urbanicity, irregular or insufficient sleep, and tobacco and alcohol in the onset of obesity. Despite the limitations of these studies (small sample size and no replication), the authors report significant findings for 12 single nucleotide polymorphisms (SNPs). Smoke modifies the interaction between *FLJ33544* and the genetic loci of olfactory pathway increasing the risk for obesity. Similarly, the combination of urban living and MC4R gene polymorphisms can raise the risk for obesity traits. Future research on the interaction between environmental factors and genetic background are essential to better unravel the pathogenic mechanisms.

Prader-Willi syndrome (PWS) is a rare genetic disorder with distinct physical, cognitive, behavioural, metabolic and endocrine symptoms (3). PWS is considered the most common syndromic form of life-threatening obesity. Despite a lower prevalence compared to non-syndromic obesity, hepatic steatosis is a common complication in PWS and may be associated with an increased risk of liver-related complications. Pascut et al. aimed to identify reliable biomarkers for the early detection of liver steatosis in adult subjects with PWS. They enrolled 31 individuals with PWS and evaluated liver steatosis through liver ultrasonography. Circulating proteome profiling was performed in 29 subjects (15 with steatosis, 14 without). The authors found 15 circulating proteins that exhibited higher expression levels in individuals with PWS and steatosis as compared with those without, with a progressive increase from grade 1 to grade 3. Interestingly, these biomarkers were significantly associated with lipid profile, transaminases, and disease-associated scores such as HOMA. They conclude that by regularly monitoring the levels of these biomarkers, healthcare professionals can detect liver steatosis at an early stage, allowing for more timely therapeutic intervention.

Musculoskeletal manifestations are commonly found in PWS, including muscle hypotonia, dysmorphic acro-facial features, scoliosis and/or kyphosis. The combination of these alterations with short stature and sarcopenia leads to impairment of motor and functional skills. In this context, Bayartai et al. first evaluated the characteristics of spinal postures and mobility in 34 adults with PWS. A non-invasive, reliable, radiation-free, computer-aided skin-surface device was used to assess spinal posture and mobility. The results were compared with those obtained from subjects with simple obesity and in adults of normal weight. They found that subjects with PWS had greater thoracic kyphosis, less lumbar lordosis, and less lumbar and hip mobility than those with normal weight. Compared to non-syndromic obesity, individuals with PWS showed reduced spinal mobility, particularly at the lumbar level. Therefore, the Authors highlight the importance of considering the peculiar characteristics of the thoracic and lumbar mobility in the management of adults with PWS.

Obesity is a recognized risk factor in the development of colorectal cancer (CC) in men, while the link between weight and CC in women is less evident (4). The study by Zhang et al. was aimed to test the genetic link between obesity and CC in females, using the GSE44076 and GSE199063 microarray datasets obtained from the Gene Expression Omnibus database. By analyzing the intersection of the two datasets, they found 146 differential genes were shared. These genes were primarily enriched in inflammatory and immune-related pathways by Gene Ontology analysis and Kyoto Encyclopedia of Genes and Genomes analysis. They also discovered 14 hub genes that were crucial in the development of CC and obesity, using protein-protein interaction building with the Cytoscape software’s MCODE and CytoNCA plug-ins. Based on these findings, this research suggests that obesity and CC are genetically linked in female subjects.

MetS is defined first by the presence of central obesity; cholelithiasis is commonly associated with obesity. The relationship between these two was examined by Zhu et al. using the experimental model of Mendelian analysis of genetic variants associated with metabolic syndrome to the presence of cholelithiasis (5). For this purpose, the Authors screened 155 SNPs strongly associated with MetS, finding a incidence of cholelithiasis, especially in subjects with obesity with high waist circumference. The study findings support the expanding evidence for the inclusion of gastrointestinal conditions within the construct of metabolic syndrome, such as fatty liver disease. Future studies examining other gastrointestinal conditions associated with central obesity are warranted, including variations of gastro-oesophageal reflux disease and its inflammatory, metaplastic variant of Barret’s oesophagitis.

It has been shown that gender and sexual hormones influence body fat distribution (6). Prompted by this evidence, Roshandel et al. investigated body fat percentage through a sex-stratified genome-wide association study. Body fat percentage was adjusted for testosterone and sex hormone binding globulin (SHBG) levels, but not their loci, to increase statistical power. They recruited 157,937 males and 154,337 females white British individuals from the UK Biobank. They identify 195 and 174 loci accounting for 3.35% and 2.60% of the variation in body fat percentage in males and females, respectively (p < 5x10^-8^). Only 38 loci were shared by both genders. These loci were not associated with cardiometabolic risk factors, and most of them were linked with favorable body fat distribution and protection from cardiometabolic disease. The authors conclude loci associated with body fat distribution adjusted for testosterone and SHBG do not show adverse cardiometabolic effects. These findings agree with the evidence that testosterone and SHBG modulate body fat distribution, exerting a favorable effect on cardiometabolic risk factors.

In conclusion, obesity is a public health concern, but genetic studies are unraveling the mechanisms that cause obesity onset and its consequences in humans. The connection between environment and genetics is a challenging aspect of these studies, but thankfully, more research is now being conducted in this area.

## Author contributions

MD: Writing – original draft. GG: Writing – original draft, Writing – review & editing. JH: Writing – review & editing.
